# Associations between cancer and Alzheimer's disease in a U.S. Medicare population

**DOI:** 10.1002/cam4.850

**Published:** 2016-09-14

**Authors:** Daryl Michal Freedman, Jincao Wu, Honglei Chen, Ralph W. Kuncl, Lindsey R. Enewold, Eric A. Engels, Neal D. Freedman, Ruth M. Pfeiffer

**Affiliations:** ^1^ Department of Health and Human ServicesNational Institutes of HealthDivision of Cancer Epidemiology and GeneticsNational Cancer InstituteRockvilleMaryland; ^2^ Aging and Neuro‐epidemiology GroupNational Institute of Environmental Health SciencesResearch Triangle ParkDurhamNorth Carolina; ^3^ Department of BiologyUniversity of RedlandsRedlandsCalifornia; ^4^ Department of Health and Human ServicesNational Institutes of HealthDivision of Cancer Control and Population SciencesNational Cancer InstituteRockvilleMaryland

**Keywords:** Alzheimer's disease, Cancer, case‐control, cohort

## Abstract

Several studies have reported bidirectional inverse associations between cancer and Alzheimer's disease (AD)**.** This study evaluates these relationships in a Medicare population. Using Surveillance, Epidemiology, and End Results (SEER) linked to Medicare data, 1992–2005, we evaluated cancer risks following AD in a case–control study of 836,947 cancer cases and 142,869 controls as well as AD risk after cancer in 742,809 cancer patients and a non‐cancer group of 420,518. We applied unconditional logistic regression to estimate odds ratios (ORs) and Cox proportional hazards models to estimate hazards ratios (HRs). We also evaluated cancer in relation to automobile injuries as a negative control to explore potential study biases. In the case–control analysis, cancer cases were less likely to have a prior diagnosis of AD than controls (OR = 0.86; 95% CI = 0.81–0.92). Cancer cases were also less likely than controls to have prior injuries from automobile accidents to the same degree (OR = 0.83; 95% CI = 0.78–0.88). In the prospective cohort, there was a lower risk observed in cancer survivors, HR = 0.87 (95% CI = 0.84–0.90). In contrast, there was no association between cancer diagnosis and subsequent automobile accident injuries (HR = 1.03; 95% CI = 0.98–1.07). That cancer risks were similarly reduced after both AD and automobile injuries suggest biases against detecting cancer in persons with unrelated medical conditions. The modestly lower AD risk in cancer survivors may reflect underdiagnosis of AD in those with a serious illness. This study does not support a relationship between cancer and AD.

## Introduction

Aging is related both to an increased risk of Alzheimer's disease (AD) and cancer, two life‐threatening diseases. Several studies have reported a bidirectional inverse association between AD and cancer such that cancer risk was lower in AD patients 1, 2, 3, 4, 5, 6, 7, 8 and vice versa 1, 2, 4, 5, 9, 10. Several biologic mechanisms have been hypothesized to underlie this inverse relationship between the two diseases, including regulation of the cell cycle, with signaling pathways regulating cell death on one hand and proliferation on the other 11, 12, 13. Molecular mechanisms have also been hypothesized; for example, the enzyme Pin1 may be overactivated in many cancers and inactivated in AD brains 12, 14, 15. Yet, whether previously observed associations reflect pathophysiologic processes rather than intrinsic limitations of epidemiologic studies remains uncertain.

An alternative explanation of the inverse association finding is ascertainment bias such that AD is less likely to be diagnosed in people with cancer, and cancer is less likely to be diagnosed in AD patients. There are special challenges in assessing the relationship between cancer and AD, as both diseases may lead to physical disabilities and AD patients are cognitively impaired. Screening or even diagnostic tests for cancer may be diminished among those already compromised by cognitive impairment 16. The intensity of medical surveillance could also influence diagnosis of cancer or AD. In addition, the relative rarity of combined cases of AD and specific cancers requires a large study population to examine associations by cancer site, including cancers which are often detected by screening.

To address these limitations and explore the relationship between AD and overall cancer as well as with specific cancer sites, we used data from a large group of Medicare patients residing within the population‐based Surveillance, Epidemiology and End‐Results (SEER) Program registry areas. We examined the risk of incident cancer after an AD diagnosis, as well as the risk of a first AD diagnosis in cancer survivors, while controlling for frequency of physician visits, a surrogate for medical surveillance. We further used a group of patients with automobile injuries as a negative control because there is no apparent biologic relationship between automobile accidents and cancer diagnoses. This allowed us to explore biases in ascertaining one serious medical condition in individuals already experiencing another.

## Materials and Methods

### Overview

We used data from Medicare, a federal health insurance system for the U.S. population aged ≥65 years, which had been linked to cancer data from the National Cancer Institute's SEER program. SEER cancer registries cover about one‐fourth of the U.S. population 17 and have a 98% cancer case ascertainment rate 18. Medicare, which includes 97% of the age‐eligible population, entitles beneficiaries to inpatient care (Part A), and 95% of those eligible subscribe to Part B, which covers physician and outpatient services 17.

The linked SEER and Medicare data provide demographic and clinical information on SEER cancer patients and their Medicare claims for services, including diagnostic codes 17. The SEER‐Medicare dataset al.so includes Medicare claims data on a 5% random beneficiary sample in SEER geographic areas. Thus, cancer cases and non‐cases represent the age‐eligible Medicare population in SEER areas 19.

### Study design

We used the SEER‐Medicare dataset for: (1) a case–control study to compute the odds of AD preceding cancer; and (2) a prospective cohort study of cancer survivors and a non‐cancer comparison cohort and the subsequent risk of AD.

**Figure 1:**
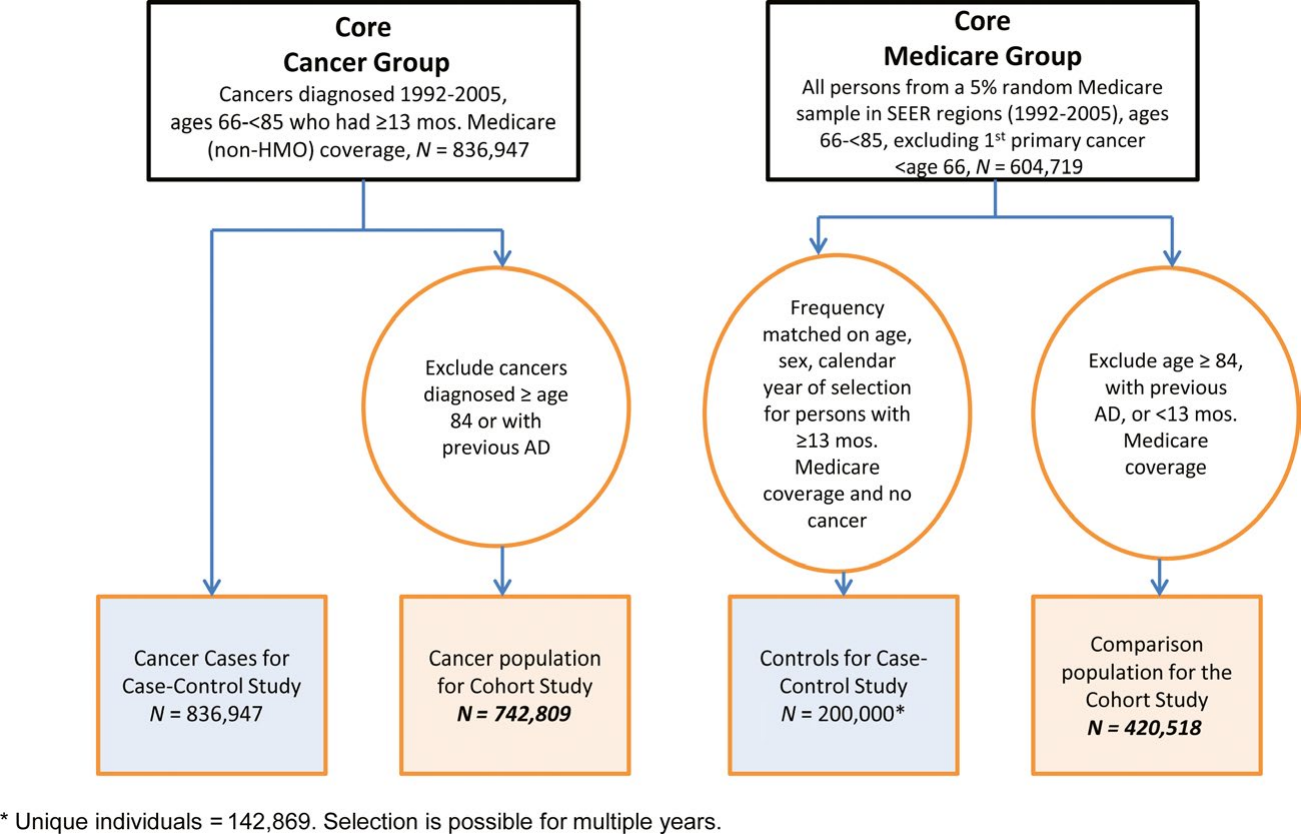
Flowchart of cancer case and non‐cancer groups.

In the case–control study, cancer cases were patients in SEER who had been diagnosed with a first primary malignancy (1992–2005). To be included, cases had at least 13 months of Medicare coverage (outside of a health maintenance organization (HMO)) prior to cancer diagnosis (with full claim information, i.e., Parts A and B), to ensure a sufficient time period to identify AD before the cancer diagnosis. HMO coverage was not counted because Medicare does not obtain claims data for enrollees in HMOs 19. Eligibility was restricted to those at least age 66 at diagnosis to permit the 13 months prior Medicare coverage, but less than age 85 because cancers (and AD) may be underascertained in the elderly due to comorbidities and short life expectancy 20. Cases were not included if diagnoses were derived from autopsy or death certificate. Total cancer cases were *N* = 836,947 (Fig. 1).

Controls for the case–control analyses were selected from the 5% Medicare sample in SEER areas (1992–2005) and were age 66–84 years old at least some time during 1992–2005, for a total of *N *= 604,719. Controls from this group were then frequency matched to cancer cases by sex, age category (5‐year intervals), and calendar year of selection if they had at least 13 months of prior Part A/Part B/non‐HMO Medicare coverage and no prior SEER‐related cancer at the time of selection. For additional details on control selection see Engels et al. 19. A total of 200,000 controls, which were sampled with replacement, were frequency matched, corresponding to *N *= 142,869 individuals (Fig. 1).

For the cohort study of AD risk after cancer diagnosis, individuals with cancer were further restricted: first, cancer cases diagnosed between ages 84 and 85 were excluded to allow at least 1 year of follow‐up after a cancer diagnosis to detect AD; and second, cases were excluded if they had been diagnosed with AD prior to study entry, yielding a total of *N *= 742,809 cancer cases in the cohort study (Fig. 1).

Similarly, subjects in the 5% random sample selected during the 1992–2005 period were eligible for inclusion in the non‐cancer comparison group if at the time of selection, they were ages 66–84 years old, had ≥13 months of Medicare coverage (Parts A/B/no HMO), and had no cancer or AD prior to selection, *N *= 420,518 (Fig. 1). The selection date for this group was the earliest date that their eligibility criteria were met.

In both the case–control and cohort studies, cancer sites were classified based on the SEER “site recode with Kaposi sarcoma and mesothelioma” variable according to International Classification of Diseases for Oncology (third edition, ICD‐O‐3). In addition to overall cancer and specific cancer sites, we grouped cancer sites into smoking‐related and non‐smoking‐related cancers because at least one study suggested the relationship between AD and cancer differed between smoking‐ and non‐smoking‐related cancers in that AD risk was lower in survivors of smoking‐related cancers 5. Smoking‐related cancer sites include cancers of the oral cavity and pharynx, lip, pancreas, lung/bronchus, larynx, cervix, kidney/renal pelvis, bladder, esophagus, and stomach;^21^ all other sites were categorized as non‐smoking‐related cancers.

AD was based on ICD‐9 code 331.0 in Medicare claims. We classified a person as having been diagnosed with AD if there was one hospital or two physician/outpatient AD claims at least 30 days apart (because hospital claims are more thoroughly audited), a method for ascertaining disease similar to that used for previous SEER‐Medicare studies of other medical conditions 19, 22.

### Statistical analyses

#### Case–control study of cancer after AD

We estimated odds ratios (ORs) and 95% confidence intervals (CIs) for the association of AD with cancer risk in cancer cases and controls using unconditional logistic regression models. We accounted for repeated selection of controls in the variance calculation 19.

#### Cohort study of AD after cancer

In the prospective cohort analysis, we compared AD incidence in cancer cases and cancer‐free individuals (comparison group). For the cancer cases, follow‐up began at the age at cancer diagnosis, and for the comparison group, at the age at selection. Follow‐up ended at the earliest age of AD diagnosis, discontinuation of Part B Medicare coverage, transfer to an HMO, death, diagnosis of a cancer (for the comparison group), attaining age 85 or December 31, 2005. With age as the time scale, we used Cox proportional hazards models to assess hazard ratios (HRs) and 95% CIs of association of AD with cancer status. We accounted for left truncation due to late age at entry using the *entrytime* statement in proc phreg, SAS 9.2.

#### Common elements to both designs and analyses

We adjusted all models for major demographic characteristics: sex, race/ethnicity (white, black, Asian‐American, Hispanic American, Native American, other), age (used as the timescale in the cohort models; in five‐year groups in the case–control models), cancer registry (because background incidences varied), and frequency of physician visits, as described below. For cohort models the baseline hazard was stratified on birth year (to account for secular trends), and case–control analyses were additionally adjusted for calendar year of cancer diagnosis/control selection (1992‐94, 1995–1998, 1999–2005), one of the matching criteria.

The first AD claim date was considered the date of AD diagnosis. AD risks were analyzed across several time intervals. In the case–control design, the intervals were <1 year; 1–<5 years; and 0–<5 years prior to cancer because Medicare claims were only available for up to 5 years for some participants. In the cohort models, the intervals were: <1 year; 1–<5 years; 5–<10 years; and 0–<10 years after cancer. Because patients with AD or cancer are often subject to heightened medical surveillance, we adjusted for frequency of physician visits, excluding claims by physicians who had limited responsibility for direct patient care, that is, radiologists, anesthesiologists, pathologists. In the case–control analyses, we adjusted for the average number of visits across all intervals (excluding the first and last). In the cohort analyses, physician visits, a maximum of one per day, were counted during 6‐month intervals between the selection and censor dates, excluding the first and last intervals. Physician visits were categorized as 0, 1–5, 6–10, >10 times during each 6‐month interval.

We assessed associations stratified by sex, race/ethnicity, and age at time of selection (66–<70; 70–<79; 80–<85 years); we also examined associations separately for specific cancer sites and whether the cancer was smoking‐related. We limited cancer site‐specific analyses (in both designs) to sites with at least 20 AD cases in the cohort analysis.

We also included analyses with all covariates other than number of physician visits and by cancer stage for several cancer sites.

We applied a Bonferroni correction to account for multiple comparisons when interpreting all the subgroups and site‐specific cancers for the total follow‐up period in the case–control and cohort analysis (*n *= 28 comparisons in each study design). This resulted in a *P*‐value of *P *<* *0.0018. *P*‐values were based on two‐sided tests.

#### Studies of cancer associated with automobile accident injuries

To evaluate potential ascertainment biases in evaluating the relationships between cancer and AD, we used automobile accident injuries (ICD‐9 E810‐819) as negative controls and conducted parallel analyses on cancer and automobile injuries. In the analysis, we simply substituted automobile accident injuries for AD in both case–control and cohort analyses. We expected the associations to be null for both designs because there is no plausible hypothesis biologically relating injuries due to automobile accidents and cancer. We also chose automobile accidents as negative controls because, like AD, a reasonable proportion are sufficiently severe 23 as to potentially impact the frequency of cancer screening/workups; accident numbers are large enough to allow comparisons between those with AD and those without AD; and there is no etiologic overlap between accidents and AD. Due to the acute nature of automobile injuries, claims for automobile accident injuries were based on a single medical visit (hospitalization or medical visit). In cohort analyses of cancer followed by automobile accident injuries, individuals were excluded if they had prior automobile accident injuries because multiple accidents may indicate a common underlying cause 24. Otherwise, the analyses were based on the models for cancer and AD.

All analyses were conducted in SAS (Version 9.2, SAS Institute, Inc., Cary, NC). This study was exempted by NIH's Office of Human Subjects Research from Institutional Review Board approval.

## Results

### Case–control analysis of Alzheimer's disease prior to cancer diagnosis

There were 836,947 cancer cases and 200,000 frequency‐matched controls in the case–control analysis of cancer after AD (Table 1). Both cancer cases and controls had a median age of 74 at diagnosis/selection and were similar across the matching variables (age, sex, selection year), as well as by race/ethnicity. In the 5 years preceding the cancer diagnoses or control selections, there were 5961 diagnoses of AD in cancer cases and 1360 in controls (Table 2).

**Table 1:** Characteristics of cancer cases and controls in retrospective case–control analysis of Alzheimer's disease prior to cancer

	Cancer cases(*N* = 836,947)	Control Group(*N* = 142,869)
Age^a^ in years, *n*(%)		
66–<70	27.0%	27.9%
70–<80	55.2%	54.7%
80–<85	17.9%	17.4%
Median age	74	74
Sex, *n*(%)		
Male	54.5%	54.8%
Female	45.5%	45.2%
Selection year, *n*(%)		
1992–1994	17.4%	17.2%
1995–1998	20.2%	20.0%
1999–2005	62.4%	62.8%
Race/ethnicity, *n*(%)		
White	86.1%	84.3%
Non‐white	13.9%	15.7%
Black	7.8%	6.7%
Asian	2.5%	3.9%
Hispanic	1.4%	2.4%
Native American Indian	0.2%	0.4%
Other/unknown	2.0%	2.3%

a For cancer cases, age is based on age at cancer diagnosis; for controls, age is based on age at selection as a control.

**Table 2:** Odds ratio (OR) for Alzheimer's disease (AD) in cancer cases compared to non‐cancer controls, 1992–2005^a^

	<1 year prior to cancer		1–<5 years prior to cancer		0–<5 years prior to cancer			
	OR	95% CI	OR	95% CI	Control^b^(*n*)	Case^b^(*n*)	OR	95% CI
Overall	0.95	0.85–1.05	0.81	0.75–0.88	1369	5961	0.86	0.81–0.92
Sex								
Men	0.91	0.78–1.06	0.74	0.65–0.84	586	2544	0.81	0.73–0.90^i^
Women	0.98	0.86–1.12	0.85	0.77–0.94	783	3417	0.90	0.82–0.98
Race								
White	0.94	0.84–1.04	0.80	0.73–0.87	1136	4888	0.85	0.79–0.91^i^
Non‐white	1.03	0.82–1.29	0.87	0.71–1.06	233	1073	0.94	0.80–1.10
Age groups (years)								
66–<70	1.06	0.70–1.59	0.90	0.61–1.31	63	299	0.96	0.72–1.28
70–<80	0.97	0.85–1.12	0.80	0.71–0.89	660	2969	0.87	0.79–0.95
80–<85	0.90	0.78–1.04	0.79	0.71–0.89	646	2693	0.84	0.76–0.92^i^
Smoking‐related cancers^c^	1.04	0.92–1.68	0.88	0.80–0.97	1369	2092	0.95	0.88–1.02
Non‐smoking‐related cancers^d^	0.91	0.82–1.01	0.77	0.71–0.84	1369	3869	0.83	0.77–0.89^i^
Individual cancer sites								
Oral cavity^e^	0.88	0.59–1.31	1.07	0.81–1.41	1369	74	0.96	0.75–1.22
Esophageal	0.84	0.55–1.29	0.89	0.64–1.23	1369	61	0.88	0.68–1.14
Stomach	1.24	0.96–1.61	1.23	1.00–1.51	1369	174	1.24	1.05–1.46
Small intestine	1.38	0.80–2.41	1.27	0.78–2.05	1369	30	1.31	0.90–1.89
Colon	1.03	0.90–1.23	0.89	0.79–1.00	1369	747	0.95	0.86–1.04
Rectum^f^	1.02	0.81–1.27	0.75	0.62–0.91	1369	204	0.84	0.72–0.98
Pancreas	1.28	1.04–1.59	1.01	0.83–1.23	1369	228	1.11	0.95–1.29
Larynx	0.52	0.26–1.01	0.55	0.32–0.93	1369	22	0.52	0.34–0.80
Lung and bronchus	1.01	0.89–1.15	0.84	0.75–0.94	1369	1036	0.91	0.84–0.91
Melanoma^g^	0.75	0.55–1.03	0.93	0.75–1.15	1136	134	0.85	0.71–1.03
Breast (female)	0.75	0.63–0.89	0.79	0.64–0.89	783	686	0.77	0.69–0.85^i^
Cervix	1.07	0.60–1.90	1.05	0.67–1.64	783	33	1.09	0.76–1.56
Uterus^h^	0.79	0.59–1.07	0.55	0.42–0.71	783	111	0.64	0.52–0.78^i^
Ovary	0.93	0.66–1.31	0.78	0.59–1.04	783	91	0.85	0.68–1.06
Prostate	0.65	0.54–0.78	0.44	0.38–0.52	586	555	0.52	0.46–0.59^i^
Bladder	1.06	0.87–1.30	0.95	0.80–1.12	1369	341	0.99	0.87–1.13
Kidney/renal pelvis	1.05	0.79–1.39	0.61	0.46–0.81	1369	111	0.78	0.64–0.96
Thyroid	0.57	0.26–1.21	0.63	0.34–1.14	1369	17	0.58	0.36–0.94
Leukemia	1.28	1.01–1.61	1.01	0.82–1.24	1369	195	1.11	0.95–1.30

a Models have been adjusted for age, race, sex, number of doctors' visits, cancer registry area, and selection years, except that sex was not adjusted for in the subpopulation based on sex, nor race, in the subpopulation defined by race. There were a total of 836,947 cancer patients and 200,000 persons in the comparison population. Data source is SEER‐Medicare. Cancer cases were classified using the “SEER site recode with Kaposi sarcoma and mesothelioma.” Refer to http://seer.cancer.gov and for details, see site recode ICD‐O‐3.

b Number of AD cases.

c Smoking‐related cancers include oral cavity and pharynx, lip, pancreas, lung and bronchus, larynx, cervix, kidney and renal pelvis, bladder, esophagus, and stomach.

d Non‐smoking‐related cancers include all cancers other than smoking‐related cancers.

e Includes tongue, floor of mouth, gum and mouth, tonsil, oropharynx, hypopharynx.

f Includes rectum and rectosigmoid junction.

g Only whites.

h Includes corpus uterine and uterus, not otherwise specified.

i *P*‐values for the associations between the groups (e.g., men) and specific cancer sites with PD (for 0 < 10 years) that were statistically significant after multiple comparison corrections (*n *= 28 comparisons) at a level of *P* < 0.0018).

Overall, cancer cases were less likely to have a prior AD diagnosis compared to controls (OR = 0.86, 95% CI = 0.81–0.92) (Table 2). The lower risk was slightly more evident in men than in women (OR = 0.81 vs. 0.90, respectively), and was largely limited to whites and participants older than age 70. Further, the inverse association with AD was more evident for non‐smoking‐related cancers than smoking‐related cancers. Adjusting for doctors’ visits attenuated the odds ratios. (Table S1). For all analyses mentioned above, the inverse association with cancer was more evident for AD identified 1–5 years before cancer diagnosis than AD identified in the year immediately before cancer diagnosis. When the 19 specific cancer sites were examined, only breast cancer (OR = 0.77), uterine cancer (OR = 0.64), and prostate cancer (OR = 0.52) were statistically significantly related to a lower odds of having a previous AD diagnosis after a Bonferroni correction (Table 2).

We also examined both the ORs for local and distant (late stage) cancer for four common cancers and did not find noticeable differences in associations for cancers of the breast, colon, and lung. Table S2. For prostate cancer, however, the risks were substantially lower for local/regional versus distant stage cancer, OR = 0.48; 95% CI = 0.41–0.55; versus OR = 0.68; 95% CI = 0.50–0.93, respectively.

In the analysis using automobile accidents as a negative control group, we found an inverse association between automobile accidents and subsequent cancer risk (OR = 0.83; 95% CI = 0.78–0.88); similar to that observed for AD and risk of subsequent cancer (Table 3), as well as inverse associations between automobile accidents and prostate cancer (OR = 0.81 (95% CI = 0.72–0.91) and breast cancer (0.86; 95% CI = 0.75–0.97).

**Table 3:** Relationship between cancer before and after injuries due to automobile accidents

HRs of injuries due to automobile accidents after cancer^a^										
	<1 year follow‐up		1–<5 years follow‐up		5–<10 years follow‐up		0–<10 years follow‐up Automobile Accident Injury Cases			
	HR	95% CI	HR	95% CI	HR	95% CI	Comparison group^c^	Cancer patients^c^	HR	95% CI
Overall	1.08	0.91–1.27	0.96	0.89–1.04	0.97	0.88–1.08	6746	6236	1.03	0.98–1.07

ORs of prior injuries due to automobile accidents in cancer cases compared to non‐cancer controls^b^								
	<1 year prior to cancer		1–<5 years prior to cancer		0–<5 years prior to cancer			
	OR	95% CI	OR	95% CI	Controls^c^	Cancer Cases^c^	OR	95% CI
Overall	0.86	0.76–0.96	0.81	0.76–0.87	1519	8403	0.83	0.78–0.88

ORs, odds ratios.

a Models have been adjusted for race, sex, and number of doctors’ visits, stratified on birth year and cancer registry area. The study populations of the cancer cohort and comparison cohort both excluded subjects with claims prior to baseline for auto accidents based on Medicare claims. Data source is SEER‐Medicare.

b Models have been adjusted for age, race, sex, number of doctors’ visits, cancer registry area, and selection years. Data source is SEER‐Medicare.

c Number of cancer cases or patients/controls or comparison group with automobile accident injuries.

### Prospective cohort analysis of Alzheimer's disease following cancer diagnosis

The prospective cohort analysis included 742,809 cancer cases and 420,518 controls at baseline and 11,812 cancer cases and 9714 controls were later identified as having AD within 10 years of follow‐up (Table 4). The cancer cases were more likely to be older, male and selected in later calendar periods, but the race/ethnicity distribution was similar in those with cancer and the comparison group. For cancer cases, there were 2.1 million person‐years of follow‐up (median 1.9 years) and for the non‐cancer comparison group, 2.4 million person‐years (median 5.8 years).

**Table 4:** Characteristics of cancer cases and non‐cancer comparison group in prospective cohort analysis of Alzheimer's disease in cancer survivors

	Cancer cases(*N *= 742,809)	Comparison Group(*N *= 420,518)
Age^a^ in years, *n*(%)		
66–<70	23.2%	64.7%
70–<80	59.9%	29.6%
80–<85	16.9%	5.7%
Median age	74	67
Sex, n (%)		
Male	55.4%	41.6%
Female	44.6%	58.4%
Selection year, *n* (%)		
1992–1994	17.8%	57.9%
1995–1998	20.3%	14.9%
1999–2005	61.9%	27.2%
Race/ethnicity, *n*(%)		
White	86.1%	83.7%
Non‐white	13.9%	16.3%
Black	7.7%	7.6%
Asian	2.5%	3.5%
Hispanic	1.4%	2.3%
Native American Indian	0.2%	0.3%
Other/unknown	2.0%	2.6%
Person‐year	2,108,469	2,435,651
Median follow‐up (yrs.)	1.9	5.8

a For cancer cases, age is based on age at cancer diagnosis; for comparison group, age is based on age at selection for the comparison group.

Overall, there was a slight statistically significant lower risk of AD in cancer cases than in the comparison group, HR = 0.87; 95% CI = 0.84–0.90 (Table 5). The inverse association was slightly stronger in women than in men; and similar to the case–control analysis of AD prior to cancer, the inverse association was largely limited to whites and older age groups. When smoking‐related cancers and non‐smoking‐related cancers were analyzed separately, the inverse association was more evident for non‐smoking‐related cancers. Before adjusting for the frequency of doctors’ visits, the HRs were more attenuated. Across all these analyses, we identified a consistent U‐shaped pattern of the temporal relationship of the association of AD with cancer: the HR was close to 1 in the first year following cancer diagnosis, fell to its lowest level in years 1–5 and then rose somewhat, but mostly retained statistical significance, 5–10 after cancer diagnosis.

**Table 5:** Hazards Ratio (HR) for Alzheimer's disease (AD) after first primary cancer diagnosis, 1992–2005^a^

	<1 year follow‐up		1–<5 year follow‐up		5–<10 year follow‐up		0–<10 year follow‐up			
	HR	95% CI	HR	95% CI	HR	95% CI	Control^b^(n)	Case^b^(n)	HR	95% CI
							
Overall	0.99	0.84–1.17	0.77	0.72–0.83	0.89	0.84–0.95	9714	11812	0.87	0.84–0.90
Sex										
Men	1.02	0.80–1.29	0.79	0.72–0.88	0.83	0.76–0.92	3126	6350	0.90	0.85–0.95^i^
Women	0.96	0.76–1.21	0.75	0.68–0.82	0.94	0.86–1.03	6588	5462	0.84	0.80–0.88^i^
Race										
White	0.97	0.81–1.17	0.75	0.70–0.81	0.90	0.84–0.97	8160	10081	0.86	0.83–0.89^i^
Non‐white	1.35	0.98–1.85	0.94	0.81–1.10	0.90	0.77–1.06	1554	1731	0.96	0.88–1.04
Age at cancer diagnosis, (years)										
66–<70	1.25	0.91–1.71	0.70	0.61–0.80	0.96	0.86–1.08	3665	1338	0.90	0.84–0.97
70–<80	0.87	0.70–1.09	0.79	0.72–0.86	0.87	0.81–0.94	8389	8193	0.87	0.83–0.90^i^
80–<84	1.05	0.75–1.49	0.78	0.66–0.91	–	–	3584	2281	0.85	0.79–0.91^i^
Smoking‐related cancers^c^	1.14	0.91–1.41	0.83	0.74–0.93	0.91	0.80–1.03	9714	2286	0.91	0.86–0.96^i^
Non‐smoking‐related cancers^d^	0.97	0.81–1.16	0.77	0.71–0.82	0.90	0.84–0.96	9714	9526	0.86	0.83–0.89^i^
Individual cancer sites										
Oral cavity^e^	1.30	0.83–2.05	0.65	0.47–0.91	0.63	0.38–1.05	9714	105	0.83	0.68–1.01
Esophageal	1.26	0.74–2.17	0.69	0.40–1.20	0.43	0.11–1.72	9714	44	0.93	0.69–1.26
Stomach	0.91	0.53–1.55	0.83	0.60–1.16	0.98	0.60–1.58	9714	100	0.83	0.67–1.02
Small intestine	0.57	0.21–1.60	0.78	0.46–1.35	0.47	0.15–1.47	9714	25	0.68	0.46–1.02
Colon	1.23	0.94–1.61	0.85	0.74–0.97	0.92	0.79–1.07	9714	1256	0.92	0.86–0.99
Rectum^f^	1.16	0.82–1.66	0.55	0.44–0.69	0.93	0.73–1.18	9714	370	0.79	0.71–0.89^i^
Pancreas	0.66	0.39–1.11	0.35	0.17–0.72	0.53	0.13–2.18	9714	45	0.68	0.50–0.92
Larynx	0.61	0.29–1.27	0.68	0.47–0.97	0.74	0.45–1.20	9714	73	0.72	0.57–0.92
Lung and bronchus	1.10	0.84–1.44	0.88	0.75–1.03	0.96	0.77–1.20	9714	791	0.96	0.88–1.04
Melanoma^g^	0.79	0.49–1.26	0.76	0.60–0.96	0.89	0.68–1.17	8160	303	0.82	0.71–0.93
Breast (female)	0.84	0.62–1.13	0.77	0.68–0.87	0.98	0.87–1.10	6588	2187	0.88	0.83–0.94^i^
Cervix	1.94	0.85–3.93	1.01	0.65–1.57	1.25	0.70–2.21	6588	64	1.30	1.00–1.69
Uterus^h^	1.04	0.68–1.58	0.63	0.50–0.78	0.84	0.67–1.05	6588	385	0.80	0.71–0.90^i^
Ovary	0.60	0.32–1.12	0.31	0.21–0.47	1.19	0.77–1.81	6588	89	0.58	0.46–0.72^i^
Prostate	0.92	0.62–1.22	0.83	0.74–0.93	0.86	0.77–0.96	3126	3574	0.90	0.85–0.96^i^
Urinary bladder	1.09	0.79–1.49	0.88	0.75–1.03	0.95	0.79–1.14	9714	779	0.97	0.89–1.06
Kidney/renal	1.27	0.85–1.89	0.79	0.62–1.00	0.84	0.61–1.14	9714	243	0.83	0.72–0.96
Thyroid	0.69	0.34–1.41	0.56	0.36–0.86	0.87	051–1.46	9717	57	0.71	0.54–0.94
Leukemia	0.79	0.50–1.26	0.64	0.48–0.84	0.71	0.48–1.05	9714	163	0.72	0.61–0.85^i^

a Models have been adjusted for race, sex and number of doctors' visits, stratified on birth year and cancer registry area, except that sex was not adjusted for in the subpopulation based on sex, nor race, in the subpopulation defined by race. There were a total of 742,809 cancer patients and 420,518 persons in the comparison population. Data source is SEER‐Medicare. Cancers were classified using the “SEER site recode with Kaposi sarcoma and mesothelioma.” Refer to http://seer.cancer.gov and for details, see site recode ICD‐O‐3.

b Number of AD cases.

c Smoking‐related cancers include oral cavity and pharynx, lip, pancreas, lung and bronchus, larynx, cervix, kidney and renal pelvis, bladder, esophagus, and stomach.

d Non‐smoking‐related cancers include all cancers other than smoking‐related cancers.

e Includes tongue, floor of mouth, gum and mouth, tonsil, oropharynx, hypopharynx.

f Includes rectum and rectosigmoid junction.

g Only whites.

h Includes corpus uterine and uterus, not otherwise specified.

i *P*‐values for the associations between the groups (e.g., men) and specific cancer sites with PD (for 0 < 10 years) that were statistically significant after multiple comparison corrections (*n *= 28 comparisons) at a level of *P* < 0.0018).

Of the 19 specific cancer sites assessed for AD risk after cancer, only six cancer sites were statistically significantly and inversely related to AD, after correction for multiple comparisons: the HRs ranged from 0.58 for ovarian cancer to 0.90 for prostate cancer and included rectal, breast, and uterine cancers and leukemia (Table 5). With the exception of cervical cancer, the HRs were smaller than 1.00 for all other cancer sites.

Unlike our analysis for cancers prior to AD diagnosis, there was no relationship between cancer and risk of subsequent injuries due to automobile accidents (HR = 1.03; 95% CI = 0.98–1.07) (Table 3).

## Discussion

In this large, population‐based SEER‐Medicare study, we found that the risk of cancer was 14% lower in AD patients and comparable to the cancer risk reduction after automobile accident injuries. The risk of AD was also 13% lower in cancer patients. In both analyses, the inverse association was largely limited to whites and participants older than age 70.

### Comparison with other studies

Several other studies have reported lower risks of AD diagnosis before and after overall cancer diagnosis 1, 2, 3, 4, 6, 7, 10, 25 or for particular cancer sites 6, 9. The findings for subpopulations, however, vary across studies. An inverse association between AD and cancer was not found in men in Ou et al.;^3^ in blacks in Roe et al. 2. (where in fact a positive association was noted); or in those <60 years in Ou et al. 3. and <65 years in Musicco et al. 4. Moreover, several studies did not find any association between AD and total cancer 9, including an autopsy study 26.

A key issue in observational epidemiologic studies of the relationship between AD and cancer is whether the observed reduced risks stem from biological factors or whether instead they reflect biases such as a lower ascertainment of cancers or AD in elderly individuals who already have a serious illness.

Several previous epidemiologic studies of the association recognize the potential impact of ascertainment bias in cognitively impaired persons 3, 6, 26, 27. Patients with dementia present difficult issues of communication and consent to testing, and may be frightened by blood draws or other screening/diagnostic interventions 28. For example, Heflin et al. found that cancer screening was less common in older persons with cognitive impairments or physical injuries 16. Also, analysis of SEER‐Medicare data showed that patients with AD were often diagnosed with cancers at more advanced stages 29, which suggests less screening and delayed comprehensive clinical evaluation. In our analyses of common cancers, we found similarly reduced risks of both local and distant cancers, although we did find markedly lower risk of local/regional than distant prostate cancer in AD patients compared to people without AD, which is consistent with reduced routine screening for prostate cancer after AD.

Our analysis of cancer ascertainment after automobile accidents supports a bias in ascertaining cancer diagnoses after AD or other serious medical conditions. We used automobile accident injuries as a negative control outcome because there is no known biological relationship between cancer and automobile injuries. In this analysis, we found that cancers were diagnosed at lower rates after AD to a similar degree as cancer after automobile accident injuries. The comparison of effect estimates of cancer risk after AD an after auto accidents is informative because health care systems may vary in the assiduousness with which they screen or work‐up those who are physically or mentally debilitated.

Moreover, the few studies that simultaneously examined the AD/cancer relationship in both directions often found lower 2, 4, sometimes substantially lower 5 risks in the analysis of cancer following AD than in the analysis of AD following cancer diagnosis. This further suggests factors other than common biologic mechanisms contributing to fewer cancers being diagnosed after AD. In addition, in our analysis of specific cancer sites, we found a similar pattern, particularly for cancers that are often detected by screening. For example, the OR for breast cancer following AD was 0.77 compared to 0.88 for AD following a breast cancer diagnosis, and corresponding risk estimates for prostate cancer was 0.52 and 0.90, respectively. Finally, we also found that the inverse association was stronger in older age groups compared to younger age groups, which is also consistent with ascertainment bias being more pronounced in the elderly who tend to have more disabilities.

Akushevich et al. 6. who evaluated AD in relation to some cancer sites in SEER‐Medicare (using different design criteria than ours) also found some significant inverse relationships but only when AD preceded cancer. While Akushevich et al. acknowledge possible biases in diagnosing cancer after AD, they argue that bias cannot fully account for the reduced risks because risks of other diseases, specifically myocardial infarction, renal disease, and ulcer, were not reduced after AD. However, the more acute symptoms associated with these conditions may make them less prone to ascertainment bias.

The inverse risks we observed for cancer after AD were also more modest than those reported by most other studies 1, 2, 4, 5, which saw up to 69% reduced risks 2. Contributing to these lower risks may be the reliance on self‐report, including postal survey;^1^, ^5^ hospitalization records 2 (rather than both inpatient and outpatient visit data); or caregiver reports 10 for cancer diagnoses, whereas the SEER/Medicare dataset uses diagnoses from cancer registries with 98% complete cancer ascertainment.

We also observed a slightly (13%) lower AD risk after a cancer diagnosis, whereas automobile accident injuries were not related to a previous cancer diagnosis. The lack of an automobile accident/cancer relationship is consistent with our expectations that there is no biologic relationship between the two conditions. Yamauchi et al. also found no relationship between cancer and subsequent externally caused injury deaths in those ≥65 years 30. Moreover, automobile injuries are likely to be detected whether or not a person has cancer and therefore are not expected to be associated with a prior cancer diagnosis. In contrast to automobile injuries, AD is a progressive chronic condition, which is often diagnosed after subjective memory complaints and comprehensive neuropsychological evaluations 31. Thus, cancer patients who are already confronting a life‐threatening condition may be less likely to be worked up for less life‐threatening conditions. Careful analysis of subpopulations may further help to evaluate potential ascertainment bias of AD in cancer survivors. As noted, the lower risk was attenuated in those <70 years. Similarly, Musico et al. 4. found no lower risk in those under age 65, as would be expected if medical evaluations for AD diagnosis were more likely in younger cancer survivors. We also observed no association in blacks and in fact risk was substantially elevated in Roe et al. 2, the only other study to examine risk by race. Health professionals may be more likely to diagnose AD in blacks once they are being seen for other medical concerns under the assumption that blacks are disproportionately burdened by AD 32. This seems to be a more plausible explanation for observed racial differences than true biological variation by race in the relationship between cancer and AD. On balance, therefore, these findings provide limited support for a biologic explanation for the reduced risk of AD following a cancer diagnosis.

### Strengths and limitations of the study

A key strength of this study is its large size, with more than 700,000 cancer cases and nearly 5 million person‐years in the cohort analysis. Whereas even the largest previous studies had less than a few hundred subjects diagnosed with both cancer and AD 4, 5, our study had nearly 12,000 cancer survivors later diagnosed with AD and nearly 6000 cancer cases who had been diagnosed with AD before their cancer diagnosis. Large numbers permitted us to assess associations with AD for specific cancer sites and for subgroups defined by race, sex, and age. As a population‐based U.S. study, this is one of only two studies that have evaluated associations by race/ethnicity 2. Because it is hypothesized that underlying biologic mechanisms contribute to an inverse relationship between cancer and AD, regardless of disease order, it is important to evaluate risks bidirectionally.

Another strength of our study is the analysis of the relationship between automobile accident injuries and cancer, which served as a comparison for evaluating potential biases in the AD/cancer relationship. Other strengths include the comprehensive ascertainment of SEER cancers; the availability of outpatient and inpatient data to identify incident AD; physician visit frequency data to control for surveillance intensity; and nationwide‐claims data for AD, which substantially mitigates losses due to migration.

Limitations of the study include the fact that Medicare studies rely on claims data, rather than validated clinical diagnoses, which could lead to underascertainment and misclassification of AD, as well as potentially incorrect assignment of dates of diagnosis. However, a study of Medicare claims data found that nearly 80% of AD patients were identified as having AD based on 5 years of claims data 33, although another study using a single year of claims found a much lower sensitivity (<40%) 34. To reduce misclassification, we identified AD cases based on both inpatient claims, which are well‐audited, and multiple outpatient/physician claims. Misclassification biases could be differential (e.g., more misclassification of, say, AD as depression among cancer survivors, which would artificially reduce risks) and/or nondifferential, which would bias toward the null. We also assumed that deaths due to conditions other than AD were independent of AD risks for those with cancer and those without cancer. Survival analysis, however, assumes this type of conditionally independent censoring.

Other limitations include the necessary restriction to Medicare‐age eligible individuals (age ≥66 years); yet, because both cancer and especially AD 35 are diseases of aging, most people with both conditions likely age‐qualify for Medicare. Also, Medicare data also lack lifestyle information, but smoking, which is most strongly related to cancer, remains of uncertain relationship with AD 35, 36, 37. In addition, our models did not account for treatment effects, which could potentially confound the relationship between the two diseases 13. Particular chemotherapeutic agents for site‐specific cancers have been linked to both neurotoxic and neuroprotective effects 13. As noted, it is also possible that some individuals with AD are misdiagnosed with depression 38. In contrast, other studies in Medicare have observed no relationship between cancer and subsequent amyotrophic lateral sclerosis 39 or Parkinson's disease 40, both diseases that have more clinically visible motor symptoms. Finally, our study's generalizability could have been affected by our exclusion of HMO subscribers, but this seems unlikely since <14% persons enrolled in HMOs during most of the study period (1996–2005), and race/age differences between the HMO and non‐HMO populations were small 41.

## Conclusions and future work

Although we found a modest inverse association between cancer and AD, the totality of the evidence supports ascertainment bias or diagnostic misclassification as an explanation for this epidemiologic observation. Therefore, this comprehensive analysis provided limited support for a true biologic relationship between cancer and AD. Future epidemiologic studies of the relationship between cancer and AD should carefully account for biases affecting identification of cancer and AD cases.

## Conflict of Interest

The authors have no conflicts of interest to disclose.

## Supporting information


**Table S1**. Relationship between cancer diagnosis before and after Alzheimer's disease (AD), adjusting for multiple variables,^a^ excluding frequency of doctors’ visits
**Table S2**. Odds ratios (ORs) for Alzheimer's disease (AD) prior to first primary cancer diagnosis, 1992‐2005, stratified by local^1^ and distant stage for selected cancers.^2^
