# Supplementary material for: Associations between cancer and Alzheimer's disease in a U.S. Medicare population
Source: Cancer Med. 2016 Sep 14;5(10):2965–76. doi: 10.1002/cam4.850 (PMC5083750; doi:10.1002/cam4.850)
Supplement: Supplementary file 1 — Table S1. Relationship between cancer diagnosis before and after Alzheimer's disease (AD), adjusting for multiple variables,a excluding frequency of doctors’ visits Table S2. Odds ratios (ORs) for Alzheimer's disease (AD) prior to first primary cancer diagnosis, 1992‐2005, stratified by local1 and distant stage for selected cancers.2 [file CAM4-5-2965-s001.docx]

Supplementary Table 1. Relationship between cancer diagnosis before and after Alzheimer’s disease (AD), adjusting for multiple variables,^a^ excluding frequency of doctors’ visits

1. ORs of AD in cancer cases compared to non-cancer controls

| 0-<5 years prior to cancer | | | | |
| --- | --- | --- | --- | --- |
|  | Controls^b^ | Cases^b^ | OR | 95% CI |
| Overall | 1369 | 5961 | 0.72 | 0.67-0.76 |
| Sex |  |  |  |  |
| Men | 586 | 2544 | 0.71 | 0.64-0.79 |
| Women | 783 | 3417 | 0.73 | 0.67-0.79 |
| Race |  |  |  |  |
| White | 1136 | 4888 | 0.70 | 0.65-0.75 |
| Non-white | 233 | 1073 | 0.80 | 0.68-0.93 |
| Age at cancer diagnosis (years) |  |  |  |  |
| 66-<70 | 63 | 299 | 0.90 | 0.67-1.19 |
| 70-<80 | 660 | 2969 | 0.74 | 0.68-0.82 |
| 80-<84 | 646 | 2693 | 0.67 | 0.61-0.74 |

1. HRs of injuries due to AD after cancer

| 0-<10 years follow-up | | | | |
| --- | --- | --- | --- | --- |
|  | Comparison group^b^ | Cases^b^ | HR | 95% CI |
| Overall | 9714 | 11812 | 0.92 | 0.89-0.95 |
| Sex |  |  |  |  |
| Men | 3126 | 6350 | 0.96 | 0.91-1.01 |
| Women | 6588 | 5462 | 0.88 | 0.84-0.92 |
| Race |  |  |  |  |
| White | 8160 | 10081 | 0.91 | 0.87-0.94 |
| Non-whites | 1554 | 1731 | 1.01 | 0.93-1.10 |
| Age at cancer diagnosis (years) |  |  |  |  |
| 66-<70 | 3665 | 1338 | 1.01 | 0.94-1.09 |
| 70-<80 | 8389 | 8193 | 0.92 | 0.88-0.95 |
| 80-<84 | 2281 | 2281 | 0.87 | 0.81-0.93 |
|  |  |  |  |  |

^a^ Case-control models (OR) have been adjusted for age, race, sex, cancer registry area, and selection years. Prospective (HR) models have been adjusted for race and sex, stratified on birth year and cancer registry area. The study populations of the cancer cohort and comparison cohort both excluded subjects with claims prior to baseline for AD based on Medicare claims. Data source is SEER-Medicare.

^b^ Number of cancer cases or controls or comparison group with AD.

Suppl Table 2. Odds ratios (ORs) for Alzheimer’s disease (AD) prior to first primary cancer diagnosis, 1992-2005, stratified by local^1^ and distant stage for selected cancers.^2^

| Selected cancers | Cancer cases diagnosed with local stage compared to controls | | Cancer cases diagnosed with distant stage compared to controls | |
| --- | --- | --- | --- | --- |
|  | OR | 95% CI | OR | 95% CI |
| Female breast | 0.68 | 0.60-0.78 | 0.64 | 0.48-0.85 |
| Prostate | 0.48 | 0.41-0.55 | 0.68 | 0.50-0.93 |
| Colon | 0.93 | 0.81-1.06 | 0.84 | 0.70-1.02 |
| Lung | 0.97 | 0.83-1.14 | 0.87 | 0.78-0.96 |

^1^ Local includes local/regional for prostate cancers.

^2^ Models adjusted for age, race, sex (except for female breast and prostate cancers), number of doctors’ visits, cancer registry and selection years. Data source is SEER-Medicare. Cancer cases were classified by using the SEER site recode with Kaposi sarcoma and mesothelioma,” Refer to http://seer.cancer.gov and for details, see site recode ICD-0-3.
